# Characterization of CADASIL among the Han Chinese in Taiwan: Distinct Genotypic and Phenotypic Profiles

**DOI:** 10.1371/journal.pone.0136501

**Published:** 2015-08-26

**Authors:** Yi-Chu Liao, Cheng-Tsung Hsiao, Jong-Ling Fuh, Chang-Ming Chern, Wei-Ju Lee, Yuh-Cherng Guo, Shuu-Jiun Wang, I-Hui Lee, Yo-Tsen Liu, Yen-Feng Wang, Feng-Chi Chang, Ming-Hung Chang, Bing-Wen Soong, Yi-Chung Lee

**Affiliations:** 1 Department of Neurology, Taipei Veterans General Hospital, Taipei, Taiwan; 2 Department of Radiology, Taipei Veterans General Hospital, Taipei, Taiwan; 3 Department of Neurology, National Yang-Ming University School of Medicine, Taipei, Taiwan; 4 Institute of Brain Science, National Yang-Ming University School of Medicine, Taipei, Taiwan; 5 Institute of Clinical Medicine, National Yang-Ming University School of Medicine, Taipei, Taiwan; 6 Brain Research Center, National Yang-Ming University, Taipei, Taiwan; 7 Department of Neurology, Taichung Veterans General Hospital, Taichung, Taiwan; 8 Department of Neurology, China Medical University Hospital, Taichung, Taiwan; 9 School of Medicine, College of Medicine, China Medical University, Taichung, Taiwan; Odense University hospital, DENMARK

## Abstract

Cerebral autosomal dominant arteriopathy with subcortical infarcts and leukoencephalopathy (CADASIL) is originally featured with a strong clustering of mutations in *NOTCH3* exons 3–6 and leukoencephalopathy with frequent anterior temporal pole involvement. The present study aims at characterizing the genotypic and phenotypic profiles of CADASIL in Taiwan. One hundred and twelve patients with CADASIL from 95 families of Chinese descents in Taiwan were identified by Sanger sequencing of exons 2 to 24 of *NOTCH3*. Twenty different mutations in *NOTCH3* were uncovered, including 3 novel ones, and R544C in exon 11 was the most common mutation, accounting for 70.5% of the pedigrees. Haplotype analyses were conducted in 14 families harboring *NOTCH3* R544C mutation and demonstrated a common haplotype linked to *NOTCH3* R544C at loci D19S929 and D19S411. Comparing with CADASIL in most Caucasian populations, CADASIL in Taiwan has several distinct features, including less frequent anterior temporal involvement, older age at symptom onset, higher incidence of intracerebral hemorrhage, and rarer occurrence of migraine. Subgroup analyses revealed that the R544C mutation is associated with lower frequency of anterior temporal involvement, later age at onset and higher frequency of cognitive dysfunction. In conclusion, the present study broadens the spectrum of *NOTCH3* mutations and provides additional insights for the clinical and molecular characteristics of CADASIL patients of Han-Chinese descents.

## Introduction

Cerebral autosomal dominant arteriopathy with subcortical infarcts and leukoencephalopathy (CADASIL) is the most common monogenic cause of ischemic stroke with characteristic manifestations of recurrent ischemic events, subcortical dementia, mood disturbance and migraine with aura [1]. It is caused by mutations in the *NOTCH3* gene (OMIM MIM*600276), which encodes a single-pass transmembrane receptor controlling cell fates during development [2]. Notch3 protein has a large extracellular region containing 34 tandem epidermal growth factor-like repeat (EGFR) domains. Each EGFR domain contains 6 cysteine residues to form three disulfide bonds that are essential for stabilizing its structure. More than 200 different mutations in *NOTCH3* have been reported worldwide (HGMD website; http://www.hgmd.cf.ac.uk/). Most of *NOTCH3* mutations are missense mutations, which usually lead to an odd number of cysteine residues and consequently a disrupted EGFR conformation [1, 3].

The spectrum of *NOTCH3* mutations varies among populations. For CADASIL populations in the United Kingdom, France and Germany, *NOTCH3* mutations most commonly occur in exon 4, followed by exon 3, 5, and 6 [3–6]. For Dutch families, exon 4 and exon 11 are the most frequently mutated exons [7]. A single *NOTCH3* mutation R544C in exon 11 accounts for the majority of CADASIL subjects in southern Korea [8, 9]. The R544C mutation was also identified in 10 out of 21 unrelated Taiwanese patients with CADASIL with a putative founder effect [10]. Interestingly, founder effects have also been reported in the Finnish families with R133C mutation as well as in the pedigrees from Veneto region of Italy with S396C mutation [11, 12]. The diverse mutational spectrums lead to differences in the optimal strategies for genetic diagnosis of CADASIL across populations. Besides, clinical phenotypes might be influenced by a distinct *NOTCH3* mutational profile and genetic background of each ethnic group. For example, CADASIL patients from East Asia have a greater risk of intracerebral hemorrhage (ICH) than those in Caucasian populations [13]. Characterization of the phenotypic and genotypic features of different populations is essential for a comprehensive understanding of CADASIL.

Although a number of studies in large series have delineated the clinical and mutational spectrums of CADASIL in European populations, the relevant data from Asia are still sparse. The present study aims at expanding the knowledge of phenotypic and genotypic features of CADASIL among the Han Chinese in Taiwan. The clinical manifestations, neuroimaging features and mutational spectrums were compared between CADASIL patients from Taiwan and those from other ethnic groups. Possible relationship between phenotypes and *NOTCH3* mutations was also elucidated.

## Materials and Methods

### Subjects

The study participants included a consecutive series of subjects with genetically confirmed CADASIL at Taipei Veterans General Hospital, a 2941-bed national medical center which serves both veterans and regular citizens in Taiwan. The initial criteria for genetic survey were subjects with marked leukoencephalopathy on neuroimaging and at least one of the following features: young age at onset of lacunar infarctions or transient ischemic attacks (TIA), cognitive dysfunction, psychiatric disorders, gait disturbance or a family history of ischemic strokes or vascular dementia. Only those with pathogenic mutations in the coding regions of *NOTCH3* were included. All participants were of Han Chinese descents. Written informed consent was obtained from each subject and the study protocol was approved by the institutional review boards of Taipei Veterans General Hospital. A total of 112 subjects from 95 families were included in the present cohort, of whom 28 subjects had been reported previously [10, 14, 15].

### Mutation detection

Genomic DNA was extracted from peripheral blood samples. Mutation analyses of exons 2 to 24 of *NOTCH3* were performed by polymerase chain reaction (PCR) amplification using intronic primers and Sanger sequencing [16]. All amplicons were sequenced for both sense and antisense strands using the Big Dye 3.1 dideoxy terminator method (Applied Biosystems, Foster City, CA) and ABI Prism 3700 Genetic Analyzer (Applied Biosystems). Mutations were identified by aligning the amplicon sequences with the published human *NOTCH3* cDNA sequence (RefSeq NM_000435.2).

### Haplotype analysis

To elucidate the founder effect of *NOTCH3* R544C mutation in Taiwan, haplotype analysis was performed in fourteen families harboring the R544C mutation as previously described [10]. In brief, six polymorphic microsatellite markers flanking *NOTCH3* and covering a region of 7.54 Kosambi cM (KcM, sex-averaged) were genotyped. These markers are D19S840, D19S929, D19S411, D19S885, D19S930, and D19S410. The first two are telomeric and the other four are centromeric to *NOTCH3*. All the information on primer sequences and allele sizes of the markers were obtained from the National Center for Biotechnology Information (NCBI) database (http://www.ncbi.nlm.nih.gov/).

### Clinical information and neuroimaging

General demographic information and clinical manifestations were obtained by patient interview or medical record review. Brain magnetic resonance imaging (MRI) scans were obtained from 96 patients with CADASIL. T2-weighed or fluid-attenuated inversion recovery (FLAIR) images of brain were reviewed retrospectively. The severity of white matter lesions (WMLs) were graded by the modified Schelten’s scale at both sides of anterior temporal pole and external capsules separately [17, 18]. The score of each region was assigned according to the followings: 0 = absent; 1 = up to five lesions < 3 mm diameter; 2 = six or more lesions < 3 mm diameter; 3 = up to five lesions 4–10 mm in diameter; 4 = six or more lesions 4–10 mm diameter; 5 = one or more lesions > 10 mm diameters; 6 = confluent hyper-intensities. Moderate or severe involvement of WMLs was defined as modified Schelten’s scale ≥ 3 on one or both sides [4, 17]. Cerebral microbleeds (CMBs) were analyzed in 32 subjects by T2-weighted gradient echo (T2*) or susceptibility-weighted angiography (SWAN) images.

### Statistical analysis

Approximately 70% of the CADASIL pedigrees in Taiwan carried the *NOTCH3* R544C mutation. To assess the genotype-phenotype relationship, the index cases were divided into subjects with the R544C mutations and those with other mutations. The age of onset and modified Schelten’s scale scores of WMLs were compared between two groups using Student’s t-test. The frequencies of clinical manifestations, lacunar infarcts, WMLs, ICHs and CMBs were compared between two groups using Chi-squared test or Fisher’s exact test. A two-sided p value <0.05 was considered to be statistically significant.

## Results

### Clinical manifestations of the participants

The demographic and clinical features of the 112 patients from 95 CADASIL pedigrees were shown in Table 1. The common initial manifestations were stroke or TIA (56.9%), followed by cognitive dysfunction (17.4%) and gait disturbance (13.8%). The average age at initial symptom onset was 54.1 ± 12.5 years (median 53.0 years). Half of the patients had one of the four cardinal symptoms of CADASIL (i.e. stroke/TIA, cognitive dysfunction, migraine or psychiatric symptoms), one third had two cardinal symptoms, and twelve patients suffered from three symptoms. Throughout the course, Stroke/TIA remained to be the most frequent manifestation of CADASIL (76.8%), followed by cognitive dysfunction (41.1%) and gait disturbance (16.1%). Migraine was present in only 2.7%, including one with aura and another two without aura. Psychiatric symptoms were noticed in 15.2% of subjects, including 2 diagnosed with schizophrenia, 11 with depression, 1 with mania and 3 with behavior changes.

**Table 1:** Demographic Data of CADASIL patients in this study.

N(%) or mean ± SD	All (N = 112)	Index case (N = 95)
Age at exam (yr; mean ± SD)	57.2 ± 12.2	58.6 ± 11.4
Age at onset (yr; mean ± SD)	54.1 ± 12.5	54.7 ± 12.4
Male	55.4%	57.9%
Family history		
Stroke	68.1%	68.2%
Cognitive dysfunction	18.9%	17.2%
Psychiatric symptoms	1.1%	1.1%
Cardiovascular risk factors		
Hypertension	39.4%	40.9%
Diabetes	14.1%	14.0%
Hyperlipidemia	18.2%	18.3%
Smoking	9.7%	6.9%
Alcohol consumption	9.6%	6.9%
Initial symptoms		
Stroke/TIA	56.9%	58.5%
Cognitive dysfunction	17.4%	18.1%
Gait disturbance, easy fallings	13.8%	16.0%
Psychiatric symptoms	3.7%	4.3%
Migraine	1.8%	1.1%
All manifestations		
Stroke/TIA	76.8%	82.1%
Cognitive dysfunction	41.1%	45.3%
Gait disturbance, easy fallings	16.1%	17.9%
Psychiatric symptoms	15.2%	16.8%
Migraine	2.7%	2.1%
MRI/MRA findings		
Lacunar infarcts (Total)	88.7%	92.9%
basal ganglion, thalamus	73.2%	78.6%
corona radiate, centrum semiovale	72.2%	77.4%
Infra-tentorium	34.8%	38.1%
Modified Schelten’s scale/presence of moderate to severe WMLs (%)		
Anterior temporal pole	2.22 ± 2.44 (44.8%)	2.39 ± 2.46 (47.0%)
External Capsule	4.18 ± 1.77 (85.4%)	4.49 ± 1.53 (90.4%)
ICH by history or image	16.2%	18.1%
CMBs by T2*/SWAN	87.5%	92.9%

Abbreviation: WMLs = white matter lesions; ICH = intracranial hemorrhage (> 1 cm); CMBs = cerebral microbleeds (<1 cm).

### Neuroimaging

**Fig 1:**
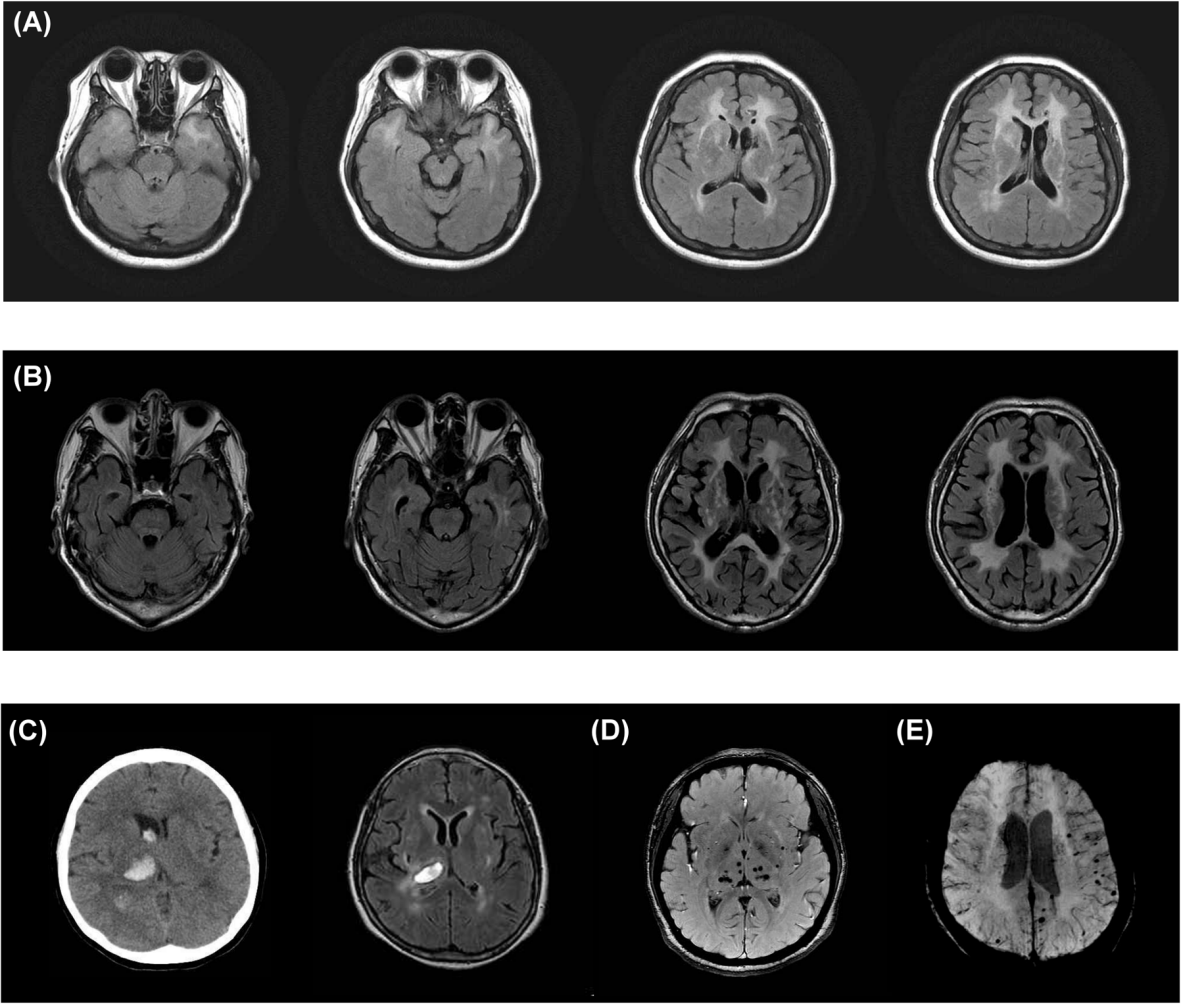
Representative neuroimaging of CADASIL patients. (A) Fluid-attenuated inversion recovery (FLAIR) images from a 54-year-old patient with *NOTCH3* R110C mutation showing diffuse white matter lesions (WMLs) with significant involvement of external capsules and anterior temporal pole. (B) FLAIR images from a 62-year-old patient with *NOTCH3* R544C mutation showing diffuse WMLs without significant involvement of anterior temporal pole. (C) Brain computer tomography and T2 weighted images from a 55-year-old patient with *NOTCH3* R544C mutation showing diffuse WMLs and an intracerebral hemorrhage (ICH) in the right thalamus on the second and eighteenth day, respectively, after the occurrence of ICH. (D) Susceptibility-weighted angiography (SWAN) images from a 48-year-old patient with *NOTCH3* R427C mutation showing multiple cerebral microbleeds in the thalami and basal ganglia. (E) SWAN images from a 53-year-old patient with *NOTCH3* R544C showing multiple cerebral microbleeds in the subcortical regions.

Brain MRI demonstrated lacunar infarcts in up to 88.7% of the patients. Deep brain structures (basal ganglia and thalami) and periventricular white matter (corona radiata and centrum semiovale) were both frequently involved (Table 1). Lacunar infarcts in the infra-tentorium regions (brainstem and cerebellum) were also detected in more than one-third of subjects. Moderate or severe WMLs in the external capsule was a common feature in 85.4% of the patients, whereas only 44.8% of the patients had significant involvement in the anterior temporal pole (Fig 1A and Fig 1B). ICH occurred in 16.2% of the subjects, including 6 ICH in the thalami, 4 in temporo-parietal region, 3 in pons, 2 in putamen, 2 in corona radiata and 1 in cerebellum (Fig 1C). CMBs were detected in 87.5% of our patients, which were most frequently observed in thalami (62.5%), followed by infra-tentorium regions (46.9%) and basal ganglia (43.8%), and least frequently in corona radiata (31.3%) (Fig 1D). CMBs were more frequently observed in CADASIL subjects with symptomatic stroke (100%) in comparison to those without stroke history (55.6%) (p = 0.004). Presence of lacunar infarctions in brain MRI was also associated with a higher risk of CMBs (p = 0.012).

### Mutational spectrum of *NOTCH3* in Taiwan

Twenty different mutations in *NOTCH3* were identified in the 95 families with CADASIL (Table 2). All the 20 mutations are cysteine-involved missense mutations. Among them, three were novel, including C224R (c.670T>C), C408Y (c.1223G>A), and C419W (c.1257C>G). Each was identified in a single patient. The clinical features and neuroimaging findings of these mutations were listed in Table 3. R544C (c.1630C>T) in exon 11 remained to be the most common mutation, accounting for 70.5% (67/95) of the pedigrees, whereas less than one fifth of the mutations (17/95) were located in exons 3 to 6. Four patients with CADASIL from 3 families carried homozygous *NOTCH3* R544C mutations. However, the clinical manifestations and age at symptom onset of R544C homozygous mutation carriers were similar to those with heterozygous mutations (Table 4).

**Table 2:** The spectrum and frequencies of *NOTCH3* mutations of CADASIL patients in this study.

*NOTCH3* mutation					
Amino acid change	coding sequence change	Exon	Domain	Index cases(N = 95)	All cases (N = 112)
C49F	c.146G>T	2	EGFR 1	1 (1.1%)	1 (0.9%)
R54C^a^	c.160C>T	2	EGFR 1	1 (1.1%)	1 (0.9%)
R90C	c.268C>T	3	EGFR 2	2 (2.1%)	2 (1.8%)
R110C	c.328C>T	3	EGFR 2	2 (2.1%)	2 (1.8%)
S118C^a^	c.353C>G	4	EGFR 2	2 (2.1%)	5 (4.5%)
R133C^a^	c 397C>T	4	EGFR 3	3 (3.2%)	3 (2.7%)
R141C^a^	c.421C>T	4	EGFR 3	2 (2.1%)	2 (1.8%)
R153C^a^	c.457C>T	4	EGFR 3	1 (1.1%)	1 (0.9%)
R169C	c.505C>T	4	EGFR 4	1 (1.1%)	1 (0.9%)
C222S	c.665C>G	4	EGFR 5	1 (1.1%)	1 (0.9%)
C224R^b^	c.670T>C	4	EGFR 5	1 (1.1%)	1 (0.9%)
R332C^a^	c.994C>T	6	EGFR 8	2 (2.1%)	2 (1.8%)
C408Y^b^	c.1223G>A	8	EGFR 10	1 (1.1%)	1 (0.9%)
C419W^b^	c.1257C>G	8	EGFR 10	1 (1.1%)	1 (0.9%)
R427C	c.1279C>T	8	EGFR 10	1 (1.1%)	1 (0.9%)
R544C^a^ ^,^ ^c^	c.1630C>T	11	EGFR 13/14	67 (70.5%)	79 (70.5%)
R587C	c.1759C>T	11	EGFR 15	1 (1.1%)	1 (0.9%)
R717C	c.2149C>T	14	EGFR 18	1 (1.1%)	1 (0.9%)
C977S^a^	c.2929T>A	18	EGFR 25	3 (3.2%)	5 (4.5%)
R1076C	c.3226C>T	20	EGFR 27	1 (1.1%)	1 (0.9%)

Epidermal growth factor-like repeats (EGFR) domain data were obtained from http://www.uniprot.org/uniprot/Q9UM47, and the R544C mutation was located at the boundary of EGFR 13 and EGFR 14, rather than within either EGFR domain.

^a^Mutations had been reported in our previous studies [10, 14, 15].

^b^Novel mutations neither have been recorded in HGMD (http://www.hgmd.cf.ac.uk/) nor reported by ExAC browser (http://exac.broadinstitute.org/gene).

^c^Three patients from 4 families carried homozygous *NOTCH3* R544C.

**Table 3:** Clinical and neuroimaging features of three novel mutations identified in the present study.

Pt/	Age at	Mutations/	ExAC	Mutation	Manifestations	MRI features			
Sex	onset	Exon	Browser	Taster		Age at MRI	Lacunar infarct	WMLs^a^	CMBs
1/F	58	C224R/4	NR	disease causing	left leg weakness (CI)	58	Bil. CR, BG	AT: 6, 6; EC: 6, 6	NA
2/M	58	C408Y/8	NR	disease causing	Unsteady gait	70	Bil. CR, BG	AT: 6, 6; EC: 6, 6	NA
3/M	78	C419W/8	NR	disease causing	dizziness, double vision (CI)	78	Bil. BG	AT: 5, 5; EC: 6, 5	NA

Abbreviation: WMLs = white matter lesions; CMBs = cerebral microbleeds; NR = not reported; CI = cerebral infarction; Bil. = bilateral; CR = corona radiata; BG = basal ganglia; AT = anterior temporal pole; EC = external capsule; NA = not available.

^a^modified Schelten’s scale of anterior temporal pole (AT) and external capsule (EC).

**Table 4:** Clinical and neuroimaging features of subjects with the R544C homozygous mutations.

Pt/	Age at	Family history		Manifestations	MRI features			
Sex	onset	Stroke	Dementia		Age at MRI	Lacunar infarct	WMLs^a^	CMBs
1/F^b^	63	+	+	unsteady gait, cognitive decline	63	Bil. CS, BG, thalami	AT: 6, 6; EC: 6, 6	NA
2/F^b^	58	+	+	repeated stroke episodes, dementia, unsteady gait,	61	Bil. CS, BG, thalami	AT: 6, 6; EC: 6, 6	NA
3/M	35	+	-	left hemiparesis, sensory deficit	35	Bil BG, CR, thalami, CS, L’t pons	AT: 1, 3; EC: 5, 5	L’t BG, R’t thalamus
4/M	52	-	-	left hemiparesis, mentality decline	53	R’t CR, BG	AT: 1, 1; EC:5, 5	NA

Abbreviation: WMLs = white matter lesions; CMBs = cerebral microbleeds; + = presence;— = absence; Bil. = bilateral; CS = centrum semiovale; BG = basal ganglia; AT = anterior temporal pole; EC = external capsule; NA = not available; CR = corona radiata; L’t = left; R’t = right.

^a^modified Schelten’s scale of anterior temporal pole (AT) and external capsule (EC)

^b^Patients 1 and 2 are sisters.

### Haplotype analysis supports the founder effect of *NOTCH3* R544C in Taiwan

**Fig 2:**
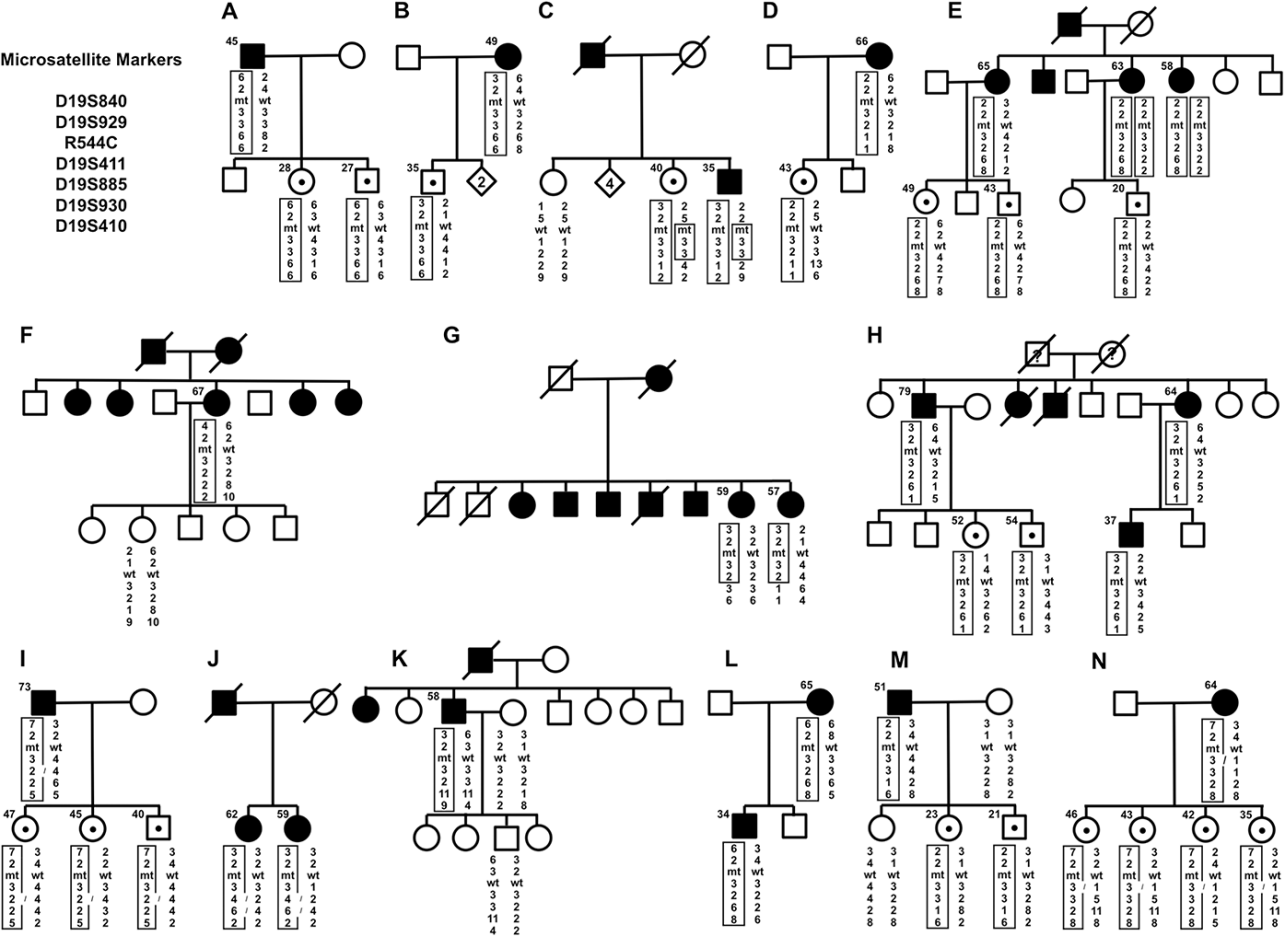
Haplotype analysis of six microsatellite markers flanking the *NOTCH3* gene in 14 CADASIL pedigrees harboring the *NOTCH3* R544C mutation. The squares and circles denote males and females with ages (years) at genetic survey on their left upper side. The filled and open symbols represent affected and unaffected members, respectively. Dotted symbols indicate asymptomatic carriers and the numbers in diamonds are the numbers of unaffected siblings. A slash indicates deceased individuals. The gender and birth order have been partially hidden for the sake of confidentiality.

Haplotype analysis was performed in 46 individuals from 14 pedigrees harboring *NOTCH3* R544C, including 21 patients, 19 asymptomatic carriers and 6 unaffected individuals. The 14 families were assigned from A to N (Fig 2), of which the haplotype analyses in families K-M had been reported previously.^10^ Thirteen families (A-M) shared a common haplotype at loci D19S929 and D19S411 linked to the *NOTCH3* R544C mutation (2-R544C-3). For those with the R544C mutation within pedigree N, although the phase of the haplotype could not be determined for sure, it was highly likely that they also had the common haplotype (2-R544C-3).

### The phenotypic differences between the CADASIL patients with R544C and those with other mutations

To determine whether *NOTCH3* R544C mutation is associated with specific features, the patients with CADASIL were divided into two groups: (1) those with the R544C mutation (n = 79) and (2) those with other mutations (n = 33). Comparison of the clinical and MRI features demonstrated significant differences in several phenotypic characteristics between these two groups (Table 5). The age at symptom onset was late for 9.1 years in the patients with R544C in comparison to subjects with other *NOTCH3* mutations (56.9 ± 12.0 and 47.8 ± 11.3 years, respectively, p = 3.6 x 10^−4^). Cognitive dysfunction was more frequently observed in the R544C group than those with other mutations (48.1% vs. 24.2%, p = 0.019). In addition, the R544C group had a family history of dementia more frequently than the other group (23.8% vs. 7.4%, p = 0.083).

**Table 5:** Comparisons between CADASIL patients with R544C or those carrying other mutations.

Features	R544C (N = 79)	Others (N = 33)	Statistics^a^
Age at onset (yr)	56.9 ± 12.0	47.8 ± 11.3	p = 3.6 x 10^−4^
Family history			
Stroke	70.3%	63.0%	p = 0.492
Dementia	23.8%	7.4%	p = 0.083
Psychiatric problems	1.6%	0.0%	p = 1.000
Clinical manifestations			
Stroke/TIA	73.4%	84.8%	p = 0.191
Cognitive dysfunction	48.1%	24.2%	p = 0.019
Psychiatric symptoms	15.2%	15.2%	p = 1.000
Migraine	3.8%	0.0%	p = 0.554
Gait disturbance	17.7%	12.1%	p = 0.579
MRI/MRA findings			
Lacunar infarcts	85.3%	96.6%	p = 0.165
Presence of moderate to severe WMLs			
Anterior temporal pole	28.4%	82.8%	p = 1.1 x 10^−6^
External Capsule	83.6%	89.7%	p = 0.542
Modified Schelten’s scale of WMLs	
Anterior temporal lobe	1.35 ± 1.93	4.22 ± 2.34	p = 1.0 x 10^−8^
External Capsule	4.10 ± 1.81	4.36 ± 1.67	P = 0.503
ICH by history or image	17.9%	12.1%	P = 0.578
CMBs by T2*/SWAN	83.3%	100.0%	P = 0.550

Abbreviation: WMLs = white matter lesions; ICH = intracranial hemorrhage; CMBs = cerebral microbleeds.

^a^Chi-squared test or Fisher’s exact test for the dichotomized variables, and Student’s t test for the continuous variables

The proportion of moderate or severe WMLs in the anterior temporal pole was significantly lower in the R544C group than that in the group with other mutations (28.4% vs. 82.8%, p = 1.1 x 10^−6^). When the severity of WMLs were quantitatively evaluated, subjects with R544C had significantly lower scores of the modified Schelten’s scales of the anterior temporal pole than those in the non-R544C group (1.35 ± 1.93 and 4.22 ± 2.34, respectively, p = 1.0 x 10^−8^), whereas the average Schelten’s scales of the external capsules were similar between the two groups (4.10 ± 1.81 and 4.36 ± 1.67, respectively, p = 0.503).

## Discussion

The present study delineates the mutational spectrum and phenotypic features of a Taiwanese cohort of 112 CADASIL patents from 95 families. In comparison with CADASIL in Caucasian populations [4, 6, 19–22], there are several distinct features of CADASIL in the Han Chinese in Taiwan. First, approximately 70% of CADASIL cases in Taiwan are resulting from the *NOTCH3* R544C mutation, whereas mutations in exon 3–6 of *NOTCH3* are responsible for approximately 90% of CADASIL patients in most Caucasian populations. Second, CADASIL in Taiwan has an older age at symptom onset (54.1 years vs. 33.6–48.5 years), higher incidence of ICH (16.2% vs. case reports) and rarer occurrence of migraine (2.7% vs. 42–75%) than those of CADASIL in most Caucasian populations. Third, Taiwanese patients have less frequent moderate or severe involvement of anterior temporal pole with WMLs than Caucasian patients with CADASIL (44.8% vs. 89%) (Table 6). Subsequent subgroup analysis revealed that CADASIL caused by the R544C mutation in Taiwan has a further later age of onset (56.9 years) and an even lower incidence of anterior temporal pole involvement of WMLs (28.4%). CADASIL patients with *NOTCH3* mutations other than R544C in Taiwan have a similar age of onset (47.8 years) and a comparable frequency of anterior temporal pole involvement (82.8%) to that of CADASIL patients in Caucasian populations (Table 6). Our study supports a correlation between specific *NOTCH3* mutations and clinical manifestations, suggesting that different mutational spectrum is responsible for the phenotype variations among ethnic groups.

**Table 6:** Phenotype-genotype correlation of CADASIL in the present study and literature reports.

Study/ populations	Total subjects/ index cases	*NOTCH3* mutations	Age of onset	Stroke*	Cognitive dysfunction	Migraine	Psychiatric symptoms	Neuroimaging
Current study, Taiwan	112/95	R544C: 70.5%	56.9 ± 12.0	73.4%	48.1%	3.8%	15.2%	AT: 28.4%, EC: 83.6%; ICH: 17.9%, CMBs: 83.3%
		Others: 29.5%	47.8 ± 11.3	84.8%	24.2%	0.0%	15.2%	AT: 82.8%, EC: 89.7%; ICH: 12.1%, CMBs: 100.0%
		Overall	54.1 ± 12.5	76.8%	41.1%	2.7%	15.2%	AT: 44.8%, EC: 85.4%; ICH: 16.2%, CMBs: 87.5%
Markus et al., UK	116/48	Exon 2–6: 93.8%; R544C: 0%; Others: 6.2%	35.9 ± 14.6 (1^st^ symptom), 43.2 (stroke)	68.8%	NA	64.6%	NA	AT: 89%, EC: 93%
Adib-Samii et al., UK	200/124	Exon 2–6: 91.9%; R544C: 0%; Others: 8.1%	33.6 ± 14.1 (1^st^ symptom), 46.0 ± 9.7 (stroke)	51.5%	16%	75.0%	37.5%	NA
Bianchi et al., Italy	229/150	Exon 2–6: 36.7%; R544C: 0%; Others: 63.3%	48.5 ± 17.1	59%	38%	42%	48%	NA
Wang et al., China	57/33	Exon 2–6: 87.7%; R544C: 0%; Others: 12.3%	42.7 ± 9.1	82.5%	59.6%	5.3%	7.0%	AT: 45.8%, EC: 100.0%
Choi et al., Korea	20/17	R544C: 75.0%	53.8 ± 7.9	66.7%	33.3%	NA	NA	ICH: 20%
		Others: 25.0%	67.2 ± 10.3	60%	80%	NA	NA	ICH: 40%
		Overall	57.2 ± 10.2	65%	45%	NA	NA	AT: 20.0%, EC: 90.0%; ICH: 25%, CMBs:73.3%
Kim et al., Korea	45/45	R544C: 27.6%	57.1 ± 9.7	85.7%	28.6%	14.3%	14.3%	AT: 42.9%, EC: 71.4%
		Others: 72.4%	52.3 ± 9.9	36.3%	50.0%	31.8%	0.0%	AT: 45.5%, EC: 77.3%
		Overall	53.4 ± 9.9	48.3%	44.8%	27.6%	3.4%	AT: 44.8%, EC: 75.9%

Abbreviation: AT = anterior temporal poles; EC = external capsules; ICH = intracranial hemorrhage; CMBs = cerebral microbleeds; NA = not available.

Number (%) in each column referred to percentage of patients presenting with corresponding manifestations (i.e. stroke, cognitive dysfunction, migraine, and psychiatric symptoms) and presence of specific neuroimaging findings (i.e. white matter lesions in AT/EC, ICH, and CMBs).

Several lines of evidence support that CADASIL with *NOTCH3* R544C mutation have particular clinical features. The R544C mutation is highly prevalent in Taiwan and Jeju island of Korea (75.0–90.3%) [8, 9], and accounts for a small proportion of CADASIL patients in mainland Korea (27.6%), China (23.1%) and Netherlands (2.3%) [7, 23, 24], but has never been identified in other populations. In the present study, the age of onset is delayed for 9.1 years in Taiwanese CADASIL subjects with R544C mutation than those with other mutations. Similarly, in Korea, a gap of 4.8 to 13.4 years of age at symptom onset was noted between CADASIL patients with the R544C mutation and those with other mutations (Table 6) [8, 23]. Moderate or severe involvement of anterior temporal pole has been considered as a useful diagnostic marker of CADASIL [4]. This idea came from the experiences of CADASIL in Caucasians. However, a lower percentage (20–45.8%) of anterior temporal pole involvement is found in CADASIL subjects from Taiwan, Korea and Mainland China [8, 13, 23]. Intriguingly, WMLs in anterior temporal pole are detected in 28.4% of Taiwanese CADASIL patients with the R544C mutation and 82.8% of the patients with other mutations. Although the reason why R544C is associated with a lower frequency of anterior temporal pole involvement remains elusive, absence of WMLs in anterior temporal pole should not be used as a standard criteria to exclude the diagnosis of CADASIL, especially in patients of East Asian origin.

The present study reveals several distinct features of CADASIL subjects with the R544C mutation, including an older age of onset, a higher percentage of cognitive dysfunction and lower frequency of anterior temporal pole involvement of WMLs (Table 5). Previous studies showed that mutations in different *NOTCH3* domains might affect vascular pathology through different mechanisms [25, 26]. Subjects with mutations retaining the signal pathway activities (EGFR 2–5) and those with mutations disrupting the ligand binding domain (EGFR 10–11) have different WMLs burden and dementia severity [26]. Besides, a younger age of stroke onset has been reported in the *NOTCH3* C174Y mutation [5], and a worse profile of WMLs has been noted in the C440G mutation [21]. All these findings support a relationship between clinical phenotypes and specific *NOTCH3* mutations. Of note, the R544C mutation occurs at the single amino acid bordered between the EGFR-13 and EGFR-14 domains of the Notch3 molecule (http://www.uniprot.org/uniprot/Q9UM47), which is different from other cysteine-involving mutations residing within an EGFR domain [1]. Therefore, comparing with other cysteine-involving *NOTCH3* mutations, R544C may result in a milder conformational change of Notch3 molecules and consequently a milder clinical phenotype.

We previously demonstrated a common haplotype at loci D19S929 and D19S311 linked to *NOTCH3* R544C mutation within three CADASIL families, which might also appear in 7 single patients with R544C [10]. This study further show that this common haplotype is present in 10 other pedigrees harboring the R544C mutation and possibly in another CADASIL family with the same mutation. These findings suggest that the Taiwanese patients carrying *NOTCH3* R544C might be descendants from a common ancestor. A founder effect has also been demonstrated for the R133C mutation in the west coast of Finland [11] and for the S396C mutation in Veneto region of Italy [12]. An older age at disease onset of CADASIL with the R544C mutation may make this mutation more easily inherited from parents to offspring than other mutations in *NOTCH3*.

It remains debatable whether this dominantly inherited disease is aggravated by homozygous mutations. In our cohort, the clinical severity and age at symptom onset of the four patients with R544C homozygous mutation were similar to those with heterozygous mutations. In literature, homozygosity of *NOTCH3* R133C mutation was reported in a 28 year-old stroke patient at the severe end of clinical spectrum whereas R578C homozygous mutation was found in a 65 year-old men with mild phenotype [27, 28]. These conflicting results failed to show a dose-dependent effect of homozygous mutations in *NOTCH3*. The rare occurrence of migraine in Taiwanese CADASIL awaits further clarification. In the present study, only three subjects were diagnosed with migraine (2.7%). In concert with our findings, Choi et al. reported the prevalence of migraine by 3.8% in Korean patients with R544C mutation [29]. Studies from mainland China and Japan also demonstrated a low prevalence of migraine in CADASIL patients ranging from 5.3% to 27.6%, regardless of *NOTCH3* mutation sites [13, 23]. This phenomenon is more likely to reflect the fact that the prevalence of migraine is significantly lower in the general population of Asians than that in the Caucasian populations [30, 31]. Spontaneous ICH has been described infrequently in Caucasian subjects with CADASIL [8, 32]. However, the prevalence of ICH in Asian patients with CADASIL is higher than expected (12.3–25.0%) [8, 9, 24]. Most of these ICHs occurred in CADASIL patients with concurrent hypertension and were located in the common sites of hypertensive ICH [8, 10],^8, 10^ suggesting that strict blood pressure control is important for CADASIL patients to avoid ICH, especially in Asian populations. The distribution and burden of CMBs have been demonstrated as a potential useful marker for the risk of symptomatic stroke [33]. Our study also reveals that CMBs were more frequently observed in symptomatic stroke group than in non-stroke group and supports the association between CMBs and symptomatic stroke.

In conclusion, we demonstrated distinct genotypic and phenotypic profiles of CADASIL in Taiwan, where *NOTCH3* R544C accounts for approximately 70% of CADASIL which is associated with an older age of onset and a lower percentage of moderate to severe WMLs in the anterior temporal pole. Three novel mutations were identified, including C224R, C408Y, and C419W. These findings broaden the spectrum of CADASIL and provide additional insights for the clinical and molecular diagnosis of CADASIL.
